# Language ideologies and the use of French in an English-dominant context of Canada: new insights into linguistic insecurity

**DOI:** 10.1515/multi-2023-0109

**Published:** 2024-01-08

**Authors:** Marie-Eve Bouchard

**Affiliations:** The University of British Columbia, Vancouver, Canada

**Keywords:** linguistic insecurity, language ideology, language policy, French-speaking schools in minority contexts, teachers, insécurité linguistique, idéologie linguistique, politique linguistique, écoles francophones en milieu minoritaire, enseignants

## Abstract

Teachers play an essential role in fostering linguistic security in their classrooms. The aim of this study is to identify the language ideologies articulated by teachers in the Francophone schools of the English-dominant context of British Columbia (Canada) in order to explore how the different practices they implement to foster the use of French in their multilingual classrooms and foster linguistic security may interact and expose contradictions. The findings are based on a thematic analysis of interviews with twenty-one French-speaking high school teachers. I argue that linguistic ideologies provide a useful locus for studying the tensions produced by institutional policies and practices and the possible impact on the students’ feelings of linguistic insecurity. Building on excerpts from the interviews, the findings indicate that the practices the teachers use to implement the French-language policy in their classrooms must be examined further as they might be harming the efforts they are making to increase linguistic security. This paper is intended to contribute to the ongoing conversation about the practical process of engaging with linguistic insecurity.

## Résumé

Les enseignants jouent un rôle essentiel dans le développement de la sécurité linguistique dans leurs salles de classe. L’objectif de cette étude est d’identifier les idéologies linguistiques articulées par les enseignants des écoles francophones de la Colombie-Britannique (province anglophone du Canada) afin d’explorer la façon dont les différentes pratiques mises en œuvre pour encourager l’utilisation du français en classe et favoriser la sécurité linguistique peuvent interagir et mettre en évidence des contradictions. Les résultats sont basés sur une analyse thématique d’entretiens avec vingt-et-un enseignants francophones du niveau secondaire. Les idéologies linguistiques fournissent un lieu idéal pour étudier les tensions produites par les politiques et les pratiques institutionnelles, ainsi que leur impact sur les sentiments d’insécurité linguistique des élèves. En s’appuyant sur des extraits des entretiens, les résultats indiquent que les pratiques utilisées par les enseignants pour mettre en œuvre la politique de la langue française dans leurs classes doivent être examinées plus en détail, car elles pourraient nuire aux efforts qu’ils déploient pour accroître la sécurité linguistique. Cet article vise à contribuer à la conversation en cours sur le processus pratique de lutte contre l’insécurité linguistique.

## 1 Introduction

Linguistic insecurity has become an important area of concern in recent years among French-speaking minorities of Canada. This insecurity, expressed as discomfort or anxiety when speaking, can lead to a loss of confidence in speaking a language, and eventually to the erosion of knowledge and ability in this language (OCOL 2021). The emergence of attitudes regarding linguistic insecurity has been attributed to school institutions, as they disseminate prestigious social norms regarding language usage, and schooling allows speakers to be aware of the distance between their ways of speaking and the prestigious forms (Francard 1993). This is why the *Fédération de la jeunesse canadienne-française* proposes to make every educational establishment in Canada a bastion of linguistic security (SNSL 2023). This implies that teachers play an essential role in fostering linguistic security in their classrooms and that their actions can exert great influence on their students.

The following study was conducted in the Canadian province of British Columbia, where the Francophone School District (pseudonym, henceforth FSD) has the mission of teaching French as a first language to ensure that students develop an appreciation of their French-speaking community, culture, and roots. This mission is definitely a challenge in the English-dominant context of British Columbia. The study focuses on linguistic security at the intersection between language ideologies (e.g., Schieffelin and Woolard 1994; Schieffelin et al. 1998) and language-in-education policy (e.g., Menken and García 2010a). More specifically, it takes interest in the beliefs the teachers hold and the practices they implement in their classrooms to encourage both linguistic security and the use of French. As Heller and Martin-Jones (2001) point out, the concept of language ideology provides a means for understanding how the beliefs and practices of teachers and discourses around language policies are shaped by hegemonic views about language. The central purpose of the study is to identify the language ideologies articulated and embodied by the teachers in order to explore how the different practices may interact and expose contradicting ideologies. The research question is: What ideologies are exhibited by teachers when discussing the practices they implement in their classrooms to encourage both linguistic security and the use of French? To answer this question, I propose to analyse the ways teachers articulate the different practices they hold to promote linguistic security and how they implement the use of French in their classroom. With this focus, I analyse interview data to identify some of the contradictions between the practices put into place to develop linguistic security and those employed to encourage the use of French. My goal as a researcher is to explore how the French-language policy is applied by teachers in their classrooms, and my goal as an activist is to collaborate with practitioners to develop the ideological spaces for promoting linguistic security within the French-speaking community of British Columbia. This is the first study on the practices that may (or may not) lead to linguistic security in the minority French-speaking communities of the English-dominant province of British Columbia. It contributes to a better understanding of linguistic insecurity by investigating the language policing practices deployed by the teachers to establish French as the medium of classroom interaction. At the end of this paper, I discuss the possible impacts of the contradicting practices and ideologies and make recommendations for the purposes of teacher education. I argue that the practices teachers use to implement the French-language policy in their classrooms must be examined further because they may be detrimental to the efforts they are making to increase linguistic security. To contextualize the study, I begin by providing background information about the French-speaking community of British Columbia.

## 2 Context: French-speaking schools in an English-dominant context of Canada

Since the early 2000s, Canadian federal policies have been prioritizing the immigration of French speakers to support the vitality of French in French-speaking minority communities outside the province of Quebec (Citizenship and Immigration Canada 2006). In his paper on the varieties of French spoken in Western Canada, Papen (2004) excluded British Columbia, arguing that the only “real” French-speaking community that existed in the province was the one in Maillardville, which has since assimilated to English. But recent work in the French-speaking communities of British Columbia suggests that the young people of today form a new cosmopolitan French-speaking community and that they are, in fact, the new “real French British Columbians” (translated from Traisnel et al. 2013: 25). The French-speaking population of British Columbia is the most culturally diverse one in the country (Adam 2003). British Columbia has a higher number of French speakers coming from abroad (32 %) and from the other Canadian provinces (56 %) than from the province itself (12 %) (OCOL 2023). The French-speaking immigrants in British Columbia come from Europe (50 %), Asia (22 %), Africa (18 %), and the Americas (10 %). From 2006 to 2016, the number of people in British Columbia who speak French as a first language at home increased by 9 %, and the number of people who speak French as the most common language increased by 21 %. Between 2012 and 2017, there was a 25 % increase in the number of students registered at French-education schools (for a total of about 40 schools and 6,000 students in 2020–2021) (OCOL 2023). In other words, the French-speaking community of British Columbia is growing. The French schools play a central role in the vitality of the French-speaking community of British Columbia, and this certainly explains why a large portion of the scholarship has focused on immigrant children in school settings (cf. Jacquet et al. 2008; Jacquet 2009; Lai-Tran 2020; Levasseur 2020).

One important area of concern among the French-speaking community of British Columbia is linguistic insecurity. To address this concern, the *Conseil jeunesse francophone de la Colombie-Britannique* (BC Francophone Youth Council, henceforth CJFCB), a not-for-profit organisation created to promote, develop, and represent the interests of the French-speaking youth of British Columbia, put together the Linguistic Security Committee. The mission of this committee is to offer interactive workshops to youth in order to raise awareness and increase linguistic security within the French-speaking community of British Columbia. The young members of the CJFCB have observed that most teachers in the FSD are from contexts where French is spoken as a majority language (mainly Quebec and France). This contrasts with the linguistic reality of British Columbia, where French is a minority language in a highly multilingual context. Therefore, the teaching staff might not be fully equipped to understand the challenges that these youth face. The members of the Linguistic Security Committee have decided to take this matter into their own hands: following the suggestion made by the *Fédération de la jeunesse canadienne-française*, they want to make every FSD school a bastion of linguistic security (CJFCB 2023). The current study is part of a broader project in collaboration with the CJFCB, which aims to expand their workshops to a broader audience – in this case, teachers – so the teachers can foster linguistic security among their students. These teachers work in FSD schools. The FSD’s mission is to 1) provide a French-language education for students attending the schools, 2) protect, promote, and transmit the French language, and 3) promote the development of a sense of belonging to French-speaking cultures. This mission clearly establishes that French is the only language of the school district, which means that teachers have to ensure that students speak French in their classrooms in order to carry out the FSD’s mission and meet the parents’ expectations. This, however, is being challenged by the students who speak English in their everyday lives, including at school.

## 3 Linguistic (in)security, language ideologies, and language policies in school

This research draws on previous studies that treat linguistic insecurity as emerging from inequalities between different social groups (Bourdieu 1982; Labov 1966). The institutionalisation of standard language varieties perpetuates what Lippi-Green (1997) called “the standard language ideology,” i.e., the belief that some varieties are more correct than others. The corrective practices that are meant to help speakers learn the so-called standard variety can have negative social consequences for their linguistic self-assurance and attitudes. One of these consequences is linguistic insecurity. Labov (1966) introduced the notion of linguistic insecurity when investigating the relationship between socioeconomic status and linguistic insecurity, which was measured with the Index of Linguistic Insecurity. Linguistic insecurity refers to speakers’ own evaluation of their variety as inferior to another variety that they perceive as “correct” or more prestigious (Meyerhoff 2006). This insecurity emerges when the speaker becomes aware of a distance between their own language variety and the variety that they consider to be legitimate (Francard 1994). According to Labov (2006: 318), “those who adopt a standard of correctness which is imposed from without, and from beyond the group which helped form their native speech pattern, are bound to show signs of linguistic insecurity.”

Since Labov (1966), a number of sociolinguists have taken interest in the causes and manifestations of linguistic insecurity, as well as ways to study this phenomenon (see Bretegnier 1999 for a review of the first decades following the work of Labov). Gueunier et al. (1978) were the first scholars to apply the notion of linguistic insecurity to the Francophone world. They investigated the use of French in terms of the standard and speaker norms, and showed that the insecurity felt by their participants related to the use of a non-standard variety. Francard’s work in the Belgian French-speaking community has made significant advances in the conceptualization of language insecurity, as he laid the foundation for a new linguistic insecurity framework. For him, linguistic insecurity is “the manifestation of a search for linguistic legitimacy, experienced by a dominated social group which has a clear perception of both the linguistic forms which show its minorization, and the language forms it needs to acquire in order to rise within the social scale” (translated from Francard 1997: 171–172 in Escandell 2011: 326). Francard replaced Labov’s original quantitative approach with a qualitative methodology, which scholars have used since then to investigate linguistic insecurity.

Most of the studies on linguistic insecurity in French-speaking communities were conducted in areas where different languages or varieties of the same language coexist (e.g., Boudreau and Dubois 1991, 1992, 1993, 2008; Francard 1989, 1993 in Belgium; Remysen 2003 in Canada; Singy 1997 in Switzerland). Calvet (1996) went a step further and extended the notion of linguistic insecurity to language contact situations – moving from an intralinguistic perspective to an interlinguistic one. Since then, a number of scholars have examined language insecurity in different multilingual contexts (e.g., Boudard 2013 in Mauritius; Bretegnier 1999 in La Réunion; Escandell 2011 in Spain; Piccardi et al. 2021 in Italy; among many others).

Studies among the French-speaking communities of Canada have shown that linguistic insecurity is an ongoing, complex, and multilayered issue, both at the individual and community levels, that tends to silence French speakers (Boudreau and Dubois 2008; Desabrais 2013; Remysen 2018). However, speakers can fight back against dominant language ideologies, assert their linguistic security, and claim the legitimacy of their language variety (Desabrais 2013). A number of studies were conducted in New Brunswick (mainly) and Ontario – the two provinces with the highest number of French speakers outside Quebec – starting in the late 1980s (e.g., Cormier and Péronnet 1987 in New Brunswick; Mougeon et al. 1984 in Ontario). At that time, French teachers in both provinces had questions about the variety they should be teaching to their students; should they teach the (non-standard) variety associated with the students’ identity, or the (standard) one that would give them access to higher education and better work opportunities? (Boudreau and Perrot 2005). Teachers were facing this dilemma as both varieties were essential for French speakers growing in a minority context. In New Brunswick, the work of Boudreau (1994, 1997, 2001, 2021 and Boudreau and Dubois (1991, 1992, 1993, 2008 is invariably referenced for all questions related to linguistic insecurity among French-speaking Acadians. The authors have shown that Acadians feel linguistically insecure, especially in regions where speakers are in everyday contact with English. And even if since the 1990s there has been a movement that valorises and normalises the use of linguistic features that are perceived as authentically Acadian, speakers do not necessarily feel comfortable with their linguistic practices (Boudreau 2021). Research on linguistic insecurity conducted among the French-speaking communities of eastern Canada is certainly relevant for the current study, but the French-speaking community of British Columbia differs from these communities that have existed and maintained French since the colonial period as it is more recent. With French speakers coming from all around the world and the everyday contact with English, linguistic (in)security in British Columbia is a fertile area for research.

Language ideology is a useful lens for examining linguistic (in)security. Language ideology is a conceptual tool used to access what speakers think, feel, and believe with regard to different languages and linguistic features (Woolard 1998). In a speech community, linguistic practices are commonly evaluated against the practices of the dominant group (Bourdieu 1982). In the case of British Columbia, as in most French-speaking minority communities of Canada, one ideological belief transmitted in the school system is that French as spoken in Quebec and France is more prestigious or that these varieties correspond to the standard form (Bouchard 2023a, 2023b). Language ideologies make the world more coherent; they arrange languages, language structures, and ways of using language as good or bad, desirable or undesirable, easy or difficult, and they link these descriptors to users of languages and language structures (Irvine 1989; Woolard 1998).

Studies of language ideologies encompass a wide range of phenomena, including linguistic insecurity. A wide range of studies focus specifically on teachers’ attitudes and beliefs towards their students’ use of languages (e.g., Huerta 2011; Pettit 2011; Sowa 2009). The classrooms are a locus for ideological work, and schools have in fact often been the focus of language ideological research (Rosa and Burdick 2017). They are dynamic sites where ideologies can be formed, reconfigured, and reinforced and where the circulating discourses can be contested or endorsed. Silverstein (1998: 138) used the notion of ideological sites to refer to these sites of institutionalized ritual and ritualization that provide a place where social groups articulate the ideological. And as Rosa and Burdick (2017: 112) wrote, “a focus on institutions can provide insights into the rituals through which language ideologies are reproduced, the social roles that participate in these processes of reproduction, and the modes of authority required for their ritual efficacy.” In this paper, I examine how the teachers’ language ideologies might (unintentionally) create or exacerbate linguistic insecurity in students. Accessing the ideologies held by teachers is key to understanding how they are used (explicitly or tacitly) to implement language policies in their classrooms, what impact they may have on students, and how they might perpetuate existing social and linguistic hierarchies.

Language education policy as a conceptual framework brings together the disciplines of linguistics and education. Because this study takes place in an English-dominant context where teachers must implement the schools’ language policy regarding the use of French, examining the interaction between language policy and classroom-level practices to implement the policy is essential. In fact, the implementation of the school language policy might have an impact on the students’ feeling of linguistic insecurity.

Language education policies are put into practice in schools around the world. But according to Menken and García (2010b), we still know little about the complex process of language policy implementation within educational contexts, and most language policy research remains national in scope rather than focusing on the central role of teachers in the classroom. Yet, as Henderson (2017: 21) puts it, “teachers are at the metaphorical heart of language policy implementation and how teachers interpret, understand, and implement language policy connects intimately with the local construction of classroom-level language policy.” Language policy encompasses all of the “language practices, beliefs and management of a community or polity” (Spolsky 2004: 9) and a number of researchers have established the importance of language ideologies in the implementation of language policy in schools and classrooms (e.g., Freeman 2004; Makoe and McKinney 2014; Palmer 2011; Rajuan and Beckerman 2011). That is the reason why this paper examines the ideologies that emerge when discussing different practices used by teachers to promote linguistic security and the use of French in their classrooms. As the school is a space often associated with the emergence of linguistic insecurity (Francard 1993), assessing how the teachers implement the use of French in their classrooms may help us understand how this may (or not) have an impact on the students’ feeling of linguistic insecurity.

## 4 Data and methods

In order to familiarise myself with the Francophone schools of British Columbia, I first made observations in one high school in Metro Vancouver. These observations were important to this study in order to understand better the teachers’ practices and to examine the students’ language choice and use in action. I participated (as an observer) in about four classes a week for a total of two months (Spring and Fall 2023), visiting classes from grade 7 to 12, with different teachers and topics. Following these observations, an e-mail was sent to all the high school teachers of the FSD to specifically introduce this research project on linguistic insecurity and to invite them to conduct an interview (with me or a research assistant) in which we would discuss their personal and professional trajectories related to teaching in French in British Columbia and the practices they apply in their classrooms to foster linguistic security and encourage the use of French. Twenty-one teachers responded positively and were interviewed. These teachers work in different schools and cities around British Columbia and they teach a broad variety of school subjects (Table 1). Semi-structured interviews in French were held online. Interviews lasted between 40 and 85 min and followed a flexible interview schedule, with questions broadly related to teaching in French in British Columbia and linguistic insecurity. The purpose of these interviews was to gather information about the teachers’ views on their students’ varieties of French, their experience as teachers in a minority context, and their classroom practices related to fostering linguistic security, and then to present this information to the CJFCB to help them create a workshop on linguistic security for teachers. This means that these interviews were not conducted with the intention of discussing language ideologies and classroom policies – but these did emerge from the analysis.

**Table 1:** Participants.

Name	Origin	Teaching subject(s)
Philippe	New Brunswick	Sciences
Sophie	France	Social sciences
Anne	British Columbia	Literature
Claudia	France	French and dramatic arts
Cassandre	Quebec	Maths and sciences
Luc	Quebec	French
Isabelle	Quebec	Social sciences
Lucas	Quebec	Physical education
Geneviève	Quebec	Maths, social justice, social sciences, Spanish
Auguste	France	French
Lisa	Quebec	Social sciences
Suzie	Quebec	French
Guillaume	Quebec	Social sciences
Caroline	British Columbia	Spanish
Alain	France	Social sciences
Pierre	France	French
Eva	Quebec	French, social sciences
Jean-Luc	British Columbia	Literature
Marie	France	Sciences
David	France	Music
Anthony	Quebec	Social sciences

Twenty hours of recordings were transcribed and anonymized. These transcribed interviews were then submitted for a thematic analysis, with the overall aim of investigating the teachers’ discursive construction of their beliefs and practices. This analysis was data-driven and consisted of grouping data into different themes that emerged during the interviews with teachers. This thematic analysis is “a method for identifying, analyzing and reporting patterns (themes) within data” (Braun and Clarke 2006: 79). Following the methodology of Braun and Clarke (2006), these different themes built a conceptual schema. These different themes will be used by the CJFCB to create a workshop on linguistic security for teachers. Among these themes, there are two that the current paper discusses in greater depth: the teachers’ perspectives on promoting linguistic security in their classrooms and their perspectives on promoting the use of French in their classrooms. This allows us to dive into the complex and contradictory nature of language ideologies and to explore the ways in which language policy implementation at the classroom level can have an impact on the students’ linguistic security.

## 5 Findings

In this section, I explore the language ideologies that emerged during the interviews. I divide the findings into two main sections: in the first section, I present the different ideologies that arose from discussing different practices used in the classroom to encourage students’ feeling of linguistic security, and in the second section, I do the same with the ideologies that arose from discussing the practices implemented to encourage the use of French in the classroom. What emerges from these different practices is a contradiction: the efforts made to support the use of French in the classroom might undermine the efforts made to cultivate linguistic security.

### 5.1 Teachers’ ideological and practical perspectives on promoting linguistic security in the classroom

#### 5.1.1 The use of English as functional

English is the only official language of British Columbia and it is the dominant language, with 94.3 % of the population speaking English as their first language (Statistics Canada 2021). Most students who attend the FSD schools speak English as (one of) their first language(s) and many feel more comfortable speaking English compared with French. English can be heard in the hallway, at lunchtime, during breaks, in the schoolyard, and even sometimes in the classrooms. This certainly contrasts with the mission of the FSD, which is to offer a French-speaking environment for students. It is partly the role of the teachers to ensure that students speak French in their classrooms, but for many, allowing students to use their English skills is a way to create an environment that is linguistically safe. This perspective represents a language ideology that the use of English is functional. It views multilingualism as a skill to be used. English has a purpose and it can assist students in developing different communication skills and learning with more ease. This ideology is reflected in the teachers’ articulation of their practices in two ways: they accept the use of English (or of English-origin words) and they themselves use English sometimes. The following two statements were identified as representing this ideology (although grounded on different arguments), which was also present in other teacher interviews:

*Quand ils parlent avec moi jamais je les pénalise, puis quand c’est hors de la salle de classe, de manière très informelle, dans les couloirs, sur des comités, des clubs, là j’autorise vraiment les anglicismes et puis ils le savent. Jamais je les reprendrais ou quoi que ce soit. S’ils disent quelque chose en anglais, c’est pas grave.* [‘When they’re talking to me, I never penalize them, but when it’s outside the classroom, in a very informal way, in the hallway, in clubs, in committees, then I really allow anglicisms and they know it. I’d never correct them or anything. If they say something in English, that’s fine.’]

(Marie)

*Comme le mot* pattern *en sciences humaines, je trouve que* pattern *en anglais est beaucoup plus beau que … je sais même plus c’est quoi le mot en français … <Interviewer : Oui des … c’est quoi des* patterns* ? (Rires)>. Des tendances* <Interviewer : OK>*, t’sais des tendances, je trouve qu’en anglais c’est plus fort avec* pattern*. […] Moi aussi il y a des mots en anglais que je préfère, il y a des choses que je préfère dire en anglais. Pis je trouve qu’il y a des mots plus forts que d’autres selon les langues. Donc des fois, je vais jouer avec la langue pour montrer qu’on peut jouer avec, pis que c’est correct, pis qu’on peut emprunter des mots*. [‘Like the word pattern in social sciences, I find that pattern in English is much more beautiful than … I don’t even know what the word is in French … <Interviewer: Yes, what are patterns? (Laughs)>. *Tendances* <Interviewer: OK> You know, *tendances*, I find that in English it’s stronger with pattern. […] For me too, there are words in English that I prefer, there are things I prefer to say in English. And I find that some words are stronger than others depending on the language. So sometimes I’ll play with the language to show that you can play with it, and that it’s OK, and that you can borrow words.’]

(Guillaume)

In the first excerpt, Marie explained that she does not “punish” or “correct” the students when they use English-origin words. For her, creating a space where it is safe for students to speak French means creating a space where it is also safe to use English. Marie does not view the use of English as a problem and chooses not to pressure students to speak French only. She allows students to use English in informal contexts, acknowledging that English plays an important role in the social lives of the students. With this approach, Marie aligns with existing recommendations to support the development of linguistic security, including honoring the students’ language skills (Remysen 2018).

In the second excerpt, Guillaume indicated that he sometimes uses English words because he finds them to be better at expressing an idea or a concept. As an example, he takes the word “pattern” and explains how the English word better conveys what he wants to say than the French translation – *tendance*. By using an English word in the classroom, Guillaume allows his students to do the same. For him, borrowing a word in such cases is acceptable. He models and shows his students how it is possible to “play with language,” i.e., to use their different language skills in a way that is conducive to the better expression of ideas. Guillaume connected the use of English with preference and better communication, promoting adherence to the ideology of English as functional.

#### 5.1.2 Mistakes as a learning tool

When asked what they do to foster linguistic security in their classrooms, a number of teachers mentioned the importance of embracing mistakes and seeing them as a learning tool. These teachers hold to the language ideology that making mistakes is a great learning opportunity and one of the most natural ways to progress. Mistake-driven learning is an ideology that emphasizes the process of learning rather than solely the results. This ideology is associated with classroom practices that aim to build resilient learners, examine the ideas of effort and persistence, and teach students to take risks. A number of teachers aim to help their students build confidence and self esteem through accepting mistakes, which at the same time creates a greater sense of linguistic security. This is explained by Jean in the following interview excerpt:

*J’insiste concrètement sur l’importance de faire des erreurs. J’insiste également sur l’importance d’essayer et j’insiste sur le fait qu’on ne juge pas les autres élèves ni moi-même pour un accent, pour une erreur, pour un mot de vocabulaire, peu importe quoi*. [‘I insist on the importance of making mistakes. I also insist on the importance of trying, and I insist on the fact that we don’t judge the other students or myself for an accent, for a mistake, for a vocabulary word, or whatever.’]

(Jean)

Making mistakes is often associated with social stigma, and the fear of making mistakes in French (or any other language) can lead to greater linguistic insecurity. This is why Jean “insists on the importance of making mistakes” and wants to bring students to value them. He also relies on his students’ non-judgemental attitude towards different ways of speaking to create a space where mistakes are welcomed. By doing so, he tries to remove the boundaries created by the fear of failure. In the following excerpt, Guillaume gives a concrete example of how a teacher can value their own mistakes, take ownership of them, and serve as a model for students:

*C’est ça, pis t’sais, des fois … « C’est quoi déjà le mot ? C’est quoi ? » Je le dis devant tout le monde : « C’est quoi le mot ? » Pis là tout le monde me regarde avec des gros yeux. Je cherche le mot, il faut que … j’essaie de ne pas le cacher. Pis je trouve ça un peu weird, t’sais dans la formation d’enseignant on dit toujours qu’il ne faut pas faire d’erreur au tableau, de fautes de français, c’est tellement … T’sais au Québec, c’est tellement comme la honte, t’as fait une faute au tableau, ça comme pas de sens, tu ne peux pas être enseignant, t’sais.* [‘That’s right, and you know, sometimes … “ What’s the word again? What’s the word?” I say it in front of everyone: “What’s the word?” And then everyone looks at me with big eyes. I’m looking for the word, I’ve got to … I’m trying not to hide it. And I find it a bit *weird*, you know, in teacher training they always say you shouldn’t make mistakes on the blackboard, French mistakes, it’s so … you know, in Quebec, it’s so like shame, you made a mistake on the blackboard, it’s unacceptable, you can’t be a teacher, you know.’]

(Guillaume)

Guillaume first explained how he normalises the fact that one might not know all the words in French and the need to ask for help (“What’s that word again?”). He does not “try to hide it” and instead he shows students how one can own the fact that they do not know a word and ask what that word is. In such cases, Guillaume is modeling; he wants to get students to speak French without worrying about making mistakes, and he shows them what to do if they do not know a word. Teachers like Jean and Guillaume aim to normalise making mistakes because in fact, most of what we learn in life comes down to trial and error. And what is good about making mistakes in a classroom setting is that not only is it possible to learn from one’s own mistakes, but also to learn from those of others. Guillaume also criticizes how teachers can be shamed for making mistakes and perceived as less competent for doing so. Jean and Guillaume’s classroom practices can be associated with a conscious normative belief about learning (i.e., mistakes are a learning tool). They demonstrated in their interviews how they reinforce this belief in the classroom by creating a space where students can make mistakes in French and by embracing their own language-related uncertainties and mistakes.

#### 5.1.3 Language variation as normal

Another ideology embraced by a number of teachers is that language variation is normal, especially among bilinguals. This ideology appears, for instance, when teachers express that code-switching is common in the speech of bilinguals – as Guillaume did above. In this sense, creating a space where alternation between French and English is welcomed is presented as an element that can foster linguistic security among students. For instance, this ideology was demonstrated by Cassandre, who showed an appreciation for code-switching strategies and who believes that we have the right to speak a language even if we do not master it perfectly: *on peut parler dans les deux langues, on peut parler même si je connais pas tous les mots en français à 100 %* (‘we can speak in both languages, we can speak even if I don’t know all the words in French 100 %’).

The ideology of language variation as normal also appears when teachers point out the importance of valuing all varieties of French – and not only the dominant varieties, which in British Columbia are the varieties from Quebec and France (Bouchard 2023a, 2023b). As presented in Table 1, a majority of the teachers come from these dominant French-speaking contexts. But students who attend the FSD schools come from a variety of linguistic backgrounds and they do not necessarily identify with the cultures and accents of Quebec and France. Different varieties of French can be heard among students. Teachers are aware of this variation, and they referred to it in the interviews as “the rainbow of the Francophonie” [*on a l’arc-en-ciel de toute la francophonie* (Geneviève)] or “a multiple Francophonie” [*une francophonie multiple* (Guillaume)]. A number of teachers have referred to the importance of offering different models of French (i.e., different accents) to foster linguistic security among students. For instance, in the interview with Lisa, she said:

*Plus ils vont avoir des modèles franco-colombiens qui sont pas, t’sais, qui sont pas franco-dominants, plus ils vont être capables de voir que, ben, c’est correct la façon dont je parle. Puis, c’est correct si je fais des erreurs à l’oral, puis, t’sais, je veux dire, l’objectif, on parlait de français standard tantôt, l’objectif, c’est d’être capable de se faire comprendre, t’sais*. [‘The more they have Franco-Colombian models who aren’t, you know, who aren’t Franco-dominant, the more they’ll be able to see that, well, the way I speak is okay. Then, it’s okay if I make mistakes when I speak, like, you know, I mean, the objective, we were talking about standard French earlier, the objective is to be understood, you know.’]

(Lisa)

Lisa considers that exposing students to different varieties of French, and especially to non-dominant varieties of French, is essential for the students to have a more positive perception of their own variety of French. Lisa also perceives errors as acceptable, and considers that being able to communicate and to be understood is the most important.

#### 5.1.4 Vulnerability as a pathway to connection and security

This ideology is also closely related to the ideology of error as a learning tool. But the focus here is rather on vulnerability: teachers choose to be vulnerable with their students and to show their insecurities in order to create an environment where failure and growth are embraced. Dale and Frye (2009) have argued that by demonstrating vulnerability, teachers choose discomfort and a growth mindset, which is what is expected from students. Being vulnerable with students means showing imperfections, weaknesses, and shortcomings, rather then shielding oneself behind a perfectly organised class, and thereby advance the growth and learning process. During the interview, a number of teachers addressed the importance of vulnerability and imperfections. In the following excerpt, Luc explains how he establishes a bond of trust with his students by showing vulnerability:

*Ben là, je viens de m’ouvrir à vous parce que ça me gêne beaucoup de parler devant vous en anglais et je l’ai fait pour vous montrer qu’il y en a qui vont vivre ça à chaque fois qu’ils vont vouloir parler en français cette année et que je vous comprends parce que je le vis à chaque fois que je parle en anglais. Donc je leur donne un peu, je me rends un peu vulnérable devant eux pour leur montrer que tout le monde … que ça se vit un peu n’importe quand et tout*. [‘Well, I’ve just opened up to you because I’m very embarrassed to speak in English in front of you, and I’ve done it to show you that there are people who are going to go through this every time they want to speak in French this year, and that I understand you because I go through this every time I speak in English. So, I give them a little, I make myself a little vulnerable in front of them to show them that everyone can go through this at any time and everything.’]

(Luc)

Luc explains how he uses vulnerability to connect with his students, bravely puts himself in an uncomfortable situation (speaking not-so-perfect English in front of his students, who mostly have English as an L1), and connects with the feeling of linguistic insecurity that many students have when they speak French. By doing so, he shows the students that it is acceptable to not speak a language perfectly. Huddy (2015) wrote that many teachers fear revealing their imperfections to their students, thinking that this might diminish their credibility. But by modeling vulnerability and showing their weaknesses – as Luc does – teachers actually provide students an accurate view of reality. In the following except, Guillaume also highlights the importance for teachers to be models and show their insecurities.

*T’sais l’enseignant qui est parfait en avant ce n’est pas accrocheur, c’est pas … T’sais, on parle de l’importance des modèles, je pense que l’enseignant peut montrer son insécurité pour agir comme modèle pour que les jeunes puissent se sentir confortables aussi par rapport à ça.* [‘You know, the teacher who’s perfect, first of all it’s not appealing … You know, we talk about the importance of role models. I think the teacher can show their insecurity to act as a role model so that young people can feel comfortable with that too.’]

(Guillaume)

Guillaume goes further by saying that not only is it important that teachers show their insecurities, but also that teachers who seem perfect are not appealing for students. Perfection allows for little to no relational connection with the students. And this sense of connection is important for students to feel safe. These three teachers highlight the value of vulnerability and kindness to create a safe space where students can be imperfect and learn, and consequently gain a greater sense of linguistic security. The four main ideologies that emerged from the discussions about the teachers’ practices in the classroom to foster linguistic security are presented and summarised in Table 2.

**Table 2:** Ideologies and practices to promote linguistic security in the classroom.

Language ideology	Interpretation	Practices
The use of English as functional	The use of English words is practical and normal in an English-dominant context; using both languages has a purpose and can assist students	Accept the use of anglicisms
		Use anglicisms
Mistakes as a learning tool	Making mistakes is normal and a way to learn	Own their mistakes
		Serve as a model for students
		Accept students’ mistakes
Language variation as normal	Language mixing is common among bilingual individuals; all varieties of French are valuable	Create a safe environment where all varieties and practices are welcomed
		Offering different models of French (different accents and varieties)
Vulnerability as a pathway to connection and security	Vulnerability is necessary to learn and develop linguistic security	Establish a bond of trust with students
		Show their own insecurities
		Be vulnerable with students

### 5.2 Teachers’ ideological and practical perspectives on promoting the use of French in the classroom

#### 5.2.1 French as the only legitimate language at school

As mentioned in Section 2, the mission of the FSD is to offer an education in French to the students who attend its schools. The responsibility of making the school a French-speaking environment mostly falls on the teachers’ shoulders. They are the ones who translate the FSD mandate into classroom practices. Some teachers (less than half) strongly believe that French has to be the only language used at school – a belief that cannot be separated from the institutional dimension of policy and ideology. To encourage the use of French in a fun way, a number of teachers organise contests. The classrooms compete against each other, starting with a certain number of points and then losing points every time a student speaks English. The understanding seems to be that English must be punished or eliminated in order to support the use of French. For instance, Isabelle (as a number of teachers) uses a point-based system in her classroom as she explains in the following excerpt:

*Et mes stratégies pour les convaincre, c’est de … je leur dis: “Ah! Moins un. Tu perds un point, c’est noté que tu parles pas en français.” […]. Après ça, j’utilise les leaders. Je le dis aux élèves qui sont francophones en premier, je leur dis qu’ils sont des leaders pis que j’ai besoin d’eux autres, qu’ils doivent m’aider. L’autre stratégie que je fais – pis ça je le fais à grandeur de l’école. Pas tout le temps, là, parce que j’ai pas tout le temps le temps. Mais c’est que quand je vois des groupes d’élèves qui parlent en anglais, soit dans ma classe ou dans le corridor, je saute, je plonge dans leur conversation, je traduis. Fait qu’ils disent … s’ils disent “Oh I went to the mall this weekend.” « Ah, tu es allé au centre d’achat! » Pis là, ils me regardent tous, pis ils sont tous … ils sont tous euh ça enlève le suivi de leur conversation, pis je dis : “Bin, continuez! Je vais continuer à-à euhm je vais continuer, moi, à vous parler en français.”* [‘And my strategies for convincing them are … I tell them: “Ah! Minus one. You lose a point, it’s noted that you didn’t speak in French.” […] After that, I use the leaders. I tell the students who are francophone first, that they’re leaders and that I need them to help me. The other strategy I use – and I use it throughout the school. Not all the time because I don’t have enough time. But when I see groups of students speaking in English, either in my classroom or in the hallway, I jump in, I dive into their conversation and I translate. If they say … if they say “Oh I went to the mall this weekend.” ‘Ah, tu es allé au centre d’achat!’ And then, they all look at me, and they’re all … they’re all … it takes away from their conversation, and I say: “Well, go on! I’ll continue to … I’ll continue to speak French.”’]

(Isabelle)

In this excerpt, Isabelle exemplifies three strategies for promoting the use of French. First, she employs a punitive, point-based system where students lose points when they do not speak French. Second, she uses the support of a few students in the classroom who are French speakers (meaning that they probably have two French-speaking parents and speak “better” French); the responsibility for implementing the use of French in the classroom then also lies with these students. Third, she jumps into group conversations in English and starts translating everything as if she were an interpreter. She does so until the students switch to French. These statements reveal how a monoglossic language ideology informs Isabelle’s practices in the classrooms, but also in the corridors. Getting the students to speak French seems to be her mission, which reveals to which extent this is a language ideology.

But some teachers (more than half of the interviewees) are more flexible than others about the use of English. For instance, teachers like Anne would prefer an approach that is more inclusive of English and that allows students to use all their language skills. But as she explained it, such an approach is not necessarily welcomed:

*J’aimerais qu’on prenne un peu plus le translanguaging en considération dans les salles de classe. You know, like, on m’avait dit … une fois, j’ai recherché des projets avec de la traduction entre l’anglais et le français, je pensais que ce serait un beau projet d’enrichissement et puis on m’a dit : “Non, il faut jamais avoir de l’anglais dans le français.” Like, il faut pas mélanger les langues.* [‘I’d like to see a little more consideration given to translanguaging in the classroom. You know, like, I was told … once, I was looking for projects involving translation between English and French, I thought it would be a nice enrichment project and then I was told: “No, you must never have English in French.” Like, you can’t mix languages.’]

(Anne)

Anne talks about translanguaging, an approach to teaching that allows and encourages students to use their full linguistic repertoire, instead of trying to stay narrowly focused on a single language (cf. García and Wei 2014; Wei 2018). But she was told that English should never be included in the classrooms and that language mixing is to be avoided. This is frustrating for Anne because she sees the use of the different languages as a way to empower students and to help them realise their full potential. Her belief contradicts the French-only ideology transmitted by the FSD and a number of teachers in the school district. She contests the imposition of the French-only ideology. In the following excerpt, another teacher, Marie, reflects on the harshness of this ideology when it is put into practice:

*[Un collègue] a dit à une élève: “Soit tu parles en français soit tu parles pas.” J’avoue je l’ai dit cette phrase aussi. Et encore je la tiens, puis je me dis que c’est horrible. On dit à un élève de se taire s’il parle pas en français, mais si ce qu’il a à dire, il sait pas le dire en français, ça veut dire qu’on l’empêche de s’exprimer. Donc c’est une phrase que je trouve vraiment dure. Voilà, je me questionne.* [‘[A colleague] said to a student, “Either you speak French or you don’t speak.” I admit that I’ve said that sentence too. And I still do, but I tell myself it’s horrible. We tell a student to shut up if they don’t speak in French, but if they can’t say what they have to say in French, that means we’re preventing them from expressing themselves. It’s a sentence I find really harsh. I’m questioning myself.’]

(Marie)

Marie admits that like her colleague, she sometimes tells students that if they do not speak French, they should not talk at all. But she realises that this ideology silences students and it undervalues an important part of their linguistic skills. It also silences an important part of their identity, as English might be their L1, their home language, or the language they speak with their friends. Marie is questioning herself, this ideology and the practices that are put into place and that end up silencing the students.

#### 5.2.2 Variable language competence and performance as normal

Perceiving variable language competence and performance as normal is an ideology that also emerged in the interviews when teachers were discussing different practices to promote the use of French in their classrooms. Many teachers recognize that variable competence and performance are common and that accepting this variability is key to creating a positive environment for the use of French (as discussed in 5.1.3 when discussing the development of linguistic security). The students who attend the French school do not necessarily have French as an L1, and sometimes not even as an L2. The FSD can enroll children of immigrant parents who have received an education in French and who have the right to a minority language education under Section 23 of the Canadian Charter of Rights and Freedom. This, of course, leads to a great range of French competency among students: some have moved to BC from France or Quebec and speak French at home with their two parents, while others speak English at home and have French as their school language only. During the interviews, teachers highlighted this variability in competency as one of the main challenges (*On n’est pas dans une réalité francophone, on est dans une réalité multilingue. Donc on a des niveaux de français qui varient beaucoup* [‘We’re not in a French-speaking reality, we’re in a multilingual reality. As a result, the levels of French vary greatly’] (Guillaume)).

The classrooms are therefore spaces that include different accents of L1 and L2 French. But the school curriculum is for speakers of L1 French; the existing material in French was created in contexts where French is dominant (i.e., Quebec and France) and it is not necessarily adapted to the level of French of some of the students. This material is not necessarily representative of their ways of speaking and their cultural contexts. Teachers have to deal with this great variability of competency and performance in their classrooms and it is partly their responsibility to ensure that students reach the level of French that is necessary to obtain their high school diploma. For the teachers, encouraging the use of French in the classrooms involves urging students to speak no matter how they speak, promoting positive French experiences, adapting the curriculum to the students’ varying levels of French, creating material that is in tune with their cultural identities and realities, and being accepting of students’ different linguistic backgrounds and levels of French proficiency. In the following excerpt, Philippe articulates how he encourages students to speak no matter how they speak, as long as they speak:

*Dans ma classe, j’encourage de parler librement. Parle-moi pas en anglais, mais parle-moi librement, puis si tu vois qu’un mot en anglais sort, c’est correct. Je comprends que tu essaies de t’exprimer.* [‘In my class, I encourage students to speak freely. Don’t speak to me in English, but speak to me freely, and then if you see that an English word comes out, that’s okay. I understand that you’re trying to express yourself.’]

(Philippe)

There is a sense of freedom around speech in this excerpt (‘to speak freely’). Philippe recognizes that students often use English when speaking. He does not want them to speak English only, but accepts the use of English words. It seems that what is most important here is communication and the effort that the students make to express themselves. In the next excerpt, Claudia also indicates that she is accepting of this variability and she sees the different levels of competency in the classroom as an opportunity to cooperate:

*Il y en a qui s’expliquent des choses en anglais parce qu’ils savent que leur ami n’est pas capable de comprendre autrement. […] mais voilà, c’est difficile de gérer un peu tout au niveau puis j’ose pas non plus travailler par groupe de niveau parce que je trouve que ça les stigmatise un peu. […] Mais en même temps, je trouve que c’est vraiment très inclusif de dire « Bah mon ami comprend mieux si je lui explique en anglais, alors je vais expliquer un petit truc en anglais », mais ça parle toujours de ce qu’on est en train de faire.* [‘Some explain things to each other in English because they know their friend can’t understand otherwise. […] but it’s difficult to manage everything at the same level, and I don’t want to work by level group either, because I think it stigmatizes them a bit. […] But at the same time, I think it’s very inclusive to say “Well, my friend understands better if I explain it in English, so I’ll explain a little something in English,” but it’s still about what we’re doing.’]

(Claudia)

Claudia sees the variable levels of competency in the classroom as an opportunity to collaborate and develop other important social skills such as communication, inclusion, supporting others, and so on. She purposefully lets students work in a group with whoever they want rather than creating groups based on their competency – which she perceives as stigmatising. The ideology that Claudia presents of variable language competence and performance as normal supports the creation of a space where students feel safe speaking French and any other languages. The two ideologies that came up in the interviews regarding the promotion of French in the classrooms are summarised in Table 3.

**Table 3:** Ideologies and practices for promoting the use of French in the classroom.

Language ideology	Interpretation	Practices
French as the only legitimate language at school	French is imposed by the school and it is the teachers’ responsibility to make the school a French-speaking environment	Contests to encourage the use of French (and punish the use of English)
	Languages have to be kept separate	
		The use of other languages is not encouraged
		Constant reminder to speak French
Variable language competence and performance as normal	The school is a space where students learn and practice French	Accepting of different language backgrounds
	Communication is key	Adapting the curriculum to the students’ level of French and interest
		Encouraging students to speak no matter how they speak, as long as they speak
		Promoting positive French experiences

## 6 Discussion and conclusion

Findings in this study suggest that the teachers’ discursive construction of their practices to cultivate linguistic security in their classrooms construes multilingualism – and more specifically the use of English – as an asset. Allowing the use of English, and therefore of the students’ broader linguistic repertoire, is perceived as a way to create a space where the students can feel safe to express themselves. Teachers accept that their students use English, and they also sometimes use English in front of their students when teaching. By doing so, they model and show the students how it is possible – and even valued – to know how to “play with language.” In such cases, the use of English is perceived as a resource that is useful for better expressing ideas. Language-mixing is also perceived as a phenomenon that characterizes bilingual speakers rather than as a problem. The teachers that hold the language ideology of language variation as normal perceive code-switching as common. They also value and appreciate the vast array of French accents and language practices in their classrooms. Because a number of students do not come from a French-speaking background, they tend to make mistakes that are different from the mistakes that students who grow up in a dominant French-speaking context would make. The ideology of seeing mistakes as a learning tool emerged from the discussions on fostering linguistic security. Ways to create a safe space for students to make mistakes and learn from them include accepting mistakes without judgement, valuing mistakes in front of the students, and modeling how one can own their mistakes and learn from them. By embracing their mistakes, teachers also choose to be vulnerable in front of their students. Establishing a bond of trust with students is essential for students to feel linguistically safe. Teachers reported developing this bond with the students by showing vulnerability and kindness. Both are necessary for learning and developing linguistic security, and teachers can lead by example by showing their own linguistic insecurities to students.

It should be mentioned that all the teachers interviewed have their students’ language security at heart. They are aware that linguistic insecurity is a problem among the French-speaking minority communities of Canada and British Columbia more specifically, and they support the development of learning tools that could support the teachers in learning how to foster linguistic security in their classrooms. However, these teachers are in a difficult position. While they value the different varieties of French spoken in their schools and appreciate the language-mixing practices that their students use, they have a responsibility as teachers to support the FSD’s mission, which includes providing a French-language education for students and promoting the use of French. This is where there is a clash at the collective level. The ideologies that emerged when discussing how to encourage French in the classroom partly diverge from the ones that came up when discussing the practices used to promote linguistic security.

A number of teachers articulated a monoglossic language ideology, which is reflected in their practices for promoting the use of French and English separately. For them, English should not be used in contexts where French is expected – including the classroom – and language-mixing practices should be corrected. This ideology contrasts with two ideologies that underpin some of the practices used to encourage linguistic security: the ideology of English as functional and the ideology of language variation as normal. One common practice where the monoglossic language ideology is manifested is in the contests that discourage the use of English or reward the use of French only. This point-based system probably advantages the students who come from a French-speaking family and disadvantages the ones from an English or multilingual background. This type of contest might work for students who thrive in competition, but could create insecurity for those who feel less confident in their French-speaking skills. Also, some teachers’ beliefs and practices align with the ideology that French is the only language to be used in school – an ideology that is central to the mission of the FSD. For those teachers, the use of French in school is presented as logical and obvious. Why would a language other than French be used in a French school? This dominant language ideology is being challenged by a number of teachers who see value in approaches that are more multilingual (such as Anne, who wanted to include translation activities) and recognize that the French-only policy might be silencing a number of students. However, even if they challenge this ideology, they cannot act differently in their classrooms because of the mandate of the FSD. This leads some of them (like Marie in the excerpt above) to questions themselves and identify their own contradictions. Finally, when discussing the use of French, the ideology of language variation as normal also emerged. For many teachers, accepting variability in the speech of students is key to promoting linguistic security, but also to promoting the use of French. They expressed a desire to hear their students speak, no matter how they speak – just as long as they speak. And with time, with more positive French-speaking experiences, these students will naturally speak more and more French.

Recent studies demonstrate that using the full language repertoire of multilingual students is beneficial for their education (e.g., García and Wei 2014). My observations in the classroom showed that these students naturally use their linguistic resources to make sense of the class content (e.g., they write in English first and then translate into French, they ask questions or for clarifications from their peers in English when they have not understood the instructions in French). This was also highlighted by Claudia, who mentioned that her students cooperate and support their peers in English when they are struggling. García and Klein (2016: 14) point out that “by recognizing bilingual students’ full language repertoire and their translanguaging capacities, we then not only improve the education of bilingual students, but, in so doing, we build a better and more just world.” I believe that an approach that is more inclusive of English in the context of British Columbia would also strengthen the students’ feeling of linguistic security and develop an appreciation of their full linguistic skills. Learning and studying in French can also mean using, maintaining, and strengthening English and other home languages.

The findings suggest that some of the practices related to the implementation of French in the classroom might be detrimental to linguistic security. It is clear that practices to promote linguistic security must be accompanied by practices that foster positive French-speaking experiences. It also seems important to continue to implement strategies that encourage the development of the full range of students’ linguistic resources. In the longer term, the FSD might also want to become more inclusive of the students’ multilingual realities.

One important limitation regarding the research methodology in this study is that the beliefs and practices the teachers shared during the interviews might not match the reality of their classroom actions (Pajares 1992), partly because these constantly change or because of the social desirability bias (Krumpal 2013) – which is the tendency for participants to respond in a way that they perceive as socially desirable. Further research on the topic could include monitoring classroom practices to see how the practices that the teachers mentioned are implemented and observe the students’ reactions. Interviews with students could also be conducted as a follow-up study so they can have their say on the different ideologies and practices presented by their teachers.
